# Lived experiences of women with chronic migraine: A phenomenological study

**DOI:** 10.1016/j.qrmh.2026.100051

**Published:** 2026-10-09

**Authors:** Mikko Patovirta, Kitty M. Suddick, Riku Nikander, Marja-Liisa Sumelahti, Pirjo Vuoskoski

**Affiliations:** a Faculty of Sport and Health Sciences, University of Jyväskylä, Jyväskylä, Finland; b School of Health Sciences, University of Brighton, Brighton, UK; c Faculty of Medicine and Health Technology, Tampere University, Tampere, Finland

**Keywords:** Chronic migraine, Embodiment, Lived experience, Body consciousness, Descriptive phenomenology

## Abstract

**Purpose:**

While quantitative research has yielded valuable insights into the prevalence, triggers, and treatment efficacy of chronic migraine, the embodied experience of the condition remains underexplored. This study portrays chronic migraine as a lived bodily condition, aiming to illuminate how such disruptions are experienced in everyday life.

**Methods:**

Data were collected from eight female participants through in-depth, open-ended phenomenological interviews. The data were analyzed using Giorgi’s descriptive phenomenological method.

**Results:**

The analysis revealed a unifying structure of the lived-through phenomenon comprising four key constituents: (1) sense of agency, (2) sense of bodily control, (3) sense of bodily exhaustion, and (4) sense of adaptation. Participants described recurring migraine attacks as involving disrupted sensations intertwined with a diminished sense of bodily control and agency. The persistent presence of pain and the anticipation of attacks were experienced as part of an ongoing state of vigilance and exhaustion. Participants expressed a tension between feeling unsafe and engaging in adaptive efforts to reconnect with their sense of agency and control.

**Conclusions:**

This study combines clinical relevance with phenomenological depth, emphasizing the subjective, pre-reflective, and partly unrecognized experiences of living with chronic migraine. Greater attention to bodily experience may support clinical encounters in which patients’ descriptions of bodily unpredictability, exhaustion, and fluctuating agency are more fully acknowledged. From a clinical perspective, attending to such experiences, as reflecting a disruption of bodily experience, may foster more responsive and person-centered approaches to rehabilitation and care in chronic migraine.

## Highlights

• Participants described a tension between feeling unsafe and adaptive efforts to reconnect with agency and bodily control.
• Persistent pain and anticipation of attacks were experienced as ongoing state of vigilance and bodily exhaustion.
• Attending to embodied experiences highlights experiential dimensions of body consciousness in migraine care.
• Understanding lived bodily experiences of chronic migraine supports a psychophysical, person-centred approach.

## Introduction

Migraine is the leading cause of disability among women aged 15–49 years and one of the leading causes of disability worldwide (Vos et al., 2020). Migraine has an estimated global prevalence of approximately 14–15% and is consistently more prevalent in women than in men (Steiner & Stovner, 2023). In Europe, an estimated 16.6% of women and 7.5% of men experience migraines (Stovner et al., 2006). According to *The International Classification of Headache Disorders* (Headache Classification Committee of the International Headache Society IHS, 2018), migraine is a highly individualized condition, with considerable variation in symptoms, triggers, and treatment responses. In addition to headaches, individuals often feel nausea, photophobia (sensitivity to light), and phonophobia (sensitivity to sound), and about one-third of migraine sufferers also report aura symptoms, which can include visual, sensory, or speech disturbances. More complex symptoms, such as brainstem or hemiplegic aura, can also occur, but are rarer (IHS, 2018). In women, hormonal fluctuations—especially the perimenstrual drop in estrogen—are linked to attack triggers (Gupta et al., 2011). The variety of symptoms, alongside pain, significantly increases the burden of the condition, adding layers of complexity and unpredictability to the migraine experience (Goadsby et al., 2017).

Chronic migraine—defined as a headache occurring on 15 or more days per month for over three months, with at least eight days meeting migraine criteria—is addressed as a particularly disabling condition (IHS, 2018). The chronic burden is often associated with medication overuse headache and coexisting conditions such as chronic pain, anxiety, depression, and obesity (Bottiroli et al., 2023, Buse et al., 2009, D’Amico et al., 2015). Individuals living with chronic migraine frequently describe a diminished quality of life and the need for ongoing management (D’Amico et al., 2015, Weatherall, 2015)). Experiences of stress overload and challenging life events, especially in the absence of effective coping strategies, are commonly reported (Buse et al., 2009, D’Amico et al., 2015, Kumar et al., 2023).

Migraine is increasingly understood as a disorder of altered sensory processing, involving mechanisms such as central sensitization and hypothalamic dysregulation, which further shape the temporal dynamics and unpredictability of attacks (Goadsby et al., 2017, Salinas-Abarca et al., 2025). Thus, it is important to distinguish chronic migraine from episodic migraine, as the former entails a more persistent and fluctuating experience of illness. Accordingly, rather than examining pain in general, this study focuses on chronic migraine as a distinct bodily experience.

Chronification is particularly burdensome because it often coincides with key life stages, including education, family formation, and career development. As migraine disproportionately affects women, it may substantially interfere in work and societal participation during these years (Steiner et al., 2020). In addition, people living with chronic migraine may express a sense of limited control over the timing of attacks or the course of their condition, which may be accompanied by feelings of helplessness (Seng et al., 2017).

While quantitative research has offered valuable insights into prevalence, triggers, mechanisms (Battista et al., 2023, Buse et al., 2009, Gao et al., 2024, Goadsby et al., 2017, Stovner et al., 2006), and treatment outcomes (Gao et al., 2024, Ha and Chu, 2024, Weatherall, 2015)), research with a specific focus on bodily meaning structures and pre-reflective embodiment in chronic migraine remains limited. A qualitative, phenomenological approach is well-suited to capturing the richness and complexity of these experiences. It allows for an exploration of how individuals perceive, describe, and make sense of their condition within the context of everyday life (Dahlberg, 2006; Giorgi, 2009, p.10–11). This phenomenological approach is also valuable from a clinical perspective. By attending to patients’ experiences, healthcare professionals may gain deeper insight into how chronic migraine is experienced in relation to daily functioning—an aspect not always visible in diagnostic assessments. Such understanding may support more empathetic, person-centered care and inform the development of approaches that resonate with patients’ actual circumstances.

A substantial and growing body of qualitative research has explored the experience of migraine, highlighting its impact on identity, daily functioning, emotional well-being, and social participation. Qualitative research by the Migraine Clinical Outcome Assessment System (MiCOAS) group has significantly advanced understanding of how migraine disrupts quality of life and everyday functioning (Mangrum et al., 2023, McGinley et al., 2025). Buse et al. (2022) and Gerstein et al. (2023) emphasize the importance of appropriate pharmacological management, whereas Mangrum et al. (2023) reported that participants valued treatments that provided consistent effects. In a subsequent study, Mangrum et al. (2024) suggested that migraine is influenced by social and contextual factors, and McGinley et al. (2025) explored migraine-related symptoms in the migraine cycle.

Research specifically focusing on the experiences of people with chronic migraine has emerged. Palacios-Ceña et al. (2017) emphasize that the patient–doctor relationship must be regarded as an important element in the strategy to improve the management of chronic migraine. In addition, a phenomenological understanding of lived experience with a migraine is established by Emmenegger and Gantenbein (2025), Battista et al. (2023), Rutberg et al. (2013), Rutberg and Öhrling (2012), and Varkey et al. (2013). Their studies highlight aspects such as fear and guilt, diminished social lives, and untrustworthy circumstances.

Several phenomenological studies have examined living with chronic migraine within a holistic framework. McPhee and Robinson (2020) found that coping with chronic migraine involves accepting both its long-term nature and the burdens it imposes. McPhee et al. (2022) suggest that primary care providers could be trained to encourage patients to shift towards accepting and managing migraines rather than endlessly hoping for a cure. Although qualitative and phenomenological research on pain and migraine has expanded, phenomenological studies have paid limited attention to the meaning structures of chronic migraine and their relationship to body consciousness.

Ontologically, this study explores the lifeworld of individuals living with chronic migraine as an embodied and intersubjective reality. Migraine is thus understood not merely as an internal pathological event, but as an intense experience situated within everyday relations to self, others, and the world. Epistemologically, the study focuses on how meanings are constituted within lived temporal consciousness, understood as the flowing structure of experience in which the present is always co-constituted by retention of the immediate past and protention of the imminent future, with the aim of revealing the essential structure shared across participants’ experiences.

## Methods

This study adopts a descriptive phenomenological approach grounded in Husserl’s philosophy (Husserl, 1982, Husserl, 1970, Husserl, 1989), Maurice Merleau-Ponty’s concept of embodiment (Merleau-Ponty, 1962), and Giorgi’s methodological framework (Giorgi, 2009). While Husserl was primarily concerned with enabling philosophical reflection (Husserl, 1960/1982), Giorgi’s descriptive method offers a rigorous approach to describing the experiences of others as they appear in the researcher’s consciousness. Husserl’s insights into temporality and consciousness support an understanding of migraine as a phenomenon unfolding over time, while Merleau-Ponty’s notion of embodiment emphasized the body as a fundamental mode of being-in-the-world, rather than merely a biological entity.

Drawing on Zahavi’s (1994) interpretation of Husserl, the body is understood as the lived body and as a center of orientation and movement in relation to which experienced objects are encountered. The body also constitutes a condition of possibility for experiencing other objects, as worldly experience is mediated through embodiment. Embodied experience is conceived as a pre-reflective mode of being in the world, structured through the kinesthetic system and the lived potentiality for movement, which is originally experienced as a spontaneous field of activity, as an “I can” (Zahavi, 1994, p. 70). Extending this view, Merleau-Ponty conceptualized the body as the “general medium for having a world,” emphasizing that embodiment is not limited to discrete sensations, but constitutes a fundamental mode of being and self-experience (Merleau-Ponty, 1962). Accordingly, in this study, “bodily experience” refers to participants' lived sensations, perceptions, bodily orientation and action, and ways of engaging with the world. From this perspective, chronic migraine may be understood as a disruption of the lived body, affecting the experiential basis of action, pre-reflective self-awareness, and everyday participation.

Giorgi’s (2009) descriptive phenomenological method is a rigorous approach to describing experiences as they manifest in consciousness, free from researcher interpretation or theoretical presuppositions. The method requires the researcher to adopt a phenomenological attitude and engage in reduction, setting aside biases and preconceived notions about the phenomenon (Giorgi, 2009, p. 98–100). Accordingly, the researcher is engaged in ongoing critical reflection, documenting presuppositions and experiential responses throughout the entire research process. In practice, this involves setting aside prior clinical and theoretical knowledge at the outset of analysis, maintaining a focus on participants’ concrete descriptions during the delineation and transformation of meaning units, and continuously ensuring that descriptions of the lived-through phenomenon remained grounded in the data. These reflections were also critically discussed with the co-authors.

Ethical approval for the study was obtained from the Ethics Committee of the Pirkanmaa Hospital District in accordance with the Declaration of Helsinki. To ensure methodological rigor, both data collection and analysis followed the principles of descriptive phenomenology (Giorgi, 2002). While the analysis aimed to remain grounded in participants' descriptions, the subsequent discussion engaged in theoretical contextualization to deepen understanding of the identified structures.

### Recruitment

Eight women aged from 21 to 65, all of whom were in regular employment and diagnosed with chronic migraine, volunteered to participate in the study (Table 1). The duration and severity of migraine symptoms experienced in everyday life varied across participants.

**Table 1:** General characteristics of the participants.

Characteristics	Description
Number of participants	8
Gender	All female
Age distribution	21–30 (n = 1), 31–45 (n = 3), 46–65 (n = 4)
Professional field	Healthcare, education, office, administration, sales
Chronic migraine diagnosis duration	< 5 years (n = 1), 5–10 years (n = 3), > 10 years (n = 4)

According to Giorgi (2009, p. 198), descriptive phenomenological research requires at least three participants to capture sufficient variation in the data and reveal the essential features of the phenomenon. Participants were recruited through a private-sector occupational healthcare provider. Healthcare professionals, including physicians, psychologists, physiotherapists, and nurses acted as gatekeepers by informing eligible clients diagnosed with chronic migraine about the study and providing them with an information sheet outlining its purpose and procedures. Interested individuals were invited to contact the first author by email. No client information was disclosed to the research team without prior consent. All participants received written information about the study and were informed of their right to withdraw at any time without providing a reason. Written informed consent was obtained prior to participation. All personal data were pseudonymized and accessible only to the first and last authors.

Individuals with severe depression were excluded because severe depression may impair the ability to recognize, attend to, and describe bodily sensations. Participants’ appointments were scheduled within a defined time frame, and they were provided with an information sheet upon registration.

### Interviews

The phenomenological in-depth interviews were conducted face-to-face and audio-recorded at the first author’s workplace between September and December, 2022. The interview process adhered to established practices within the phenomenological tradition (Bevan, 2014, Englander, 2012). According to Englander (2012), interviewing from a phenomenological perspective requires shifting presence within a single mode of consciousness. Furthermore, phenomenological interviewing requires the interviewer to balance the subject–subject relation, in which attention is given to the participant's account, with the subject–phenomenon relation, in which questions are guided by the research phenomenon (Englander, 2012). While listening attentively to the participant, the interviewer must identify which aspects of the described experience require further clarification to illuminate the structure of the experience (Bevan, 2014).

Two primary questions were asked: (1) Could you describe your lived bodily migraine experience in your own words, in as much detail as possible? (2) Could you describe, in your own words and in as much detail as possible, your lived bodily experience during a single migraine attack? The term “bodily experience” was used in an open, non-defining manner to encourage participants to describe their experiences in their own terms, without imposing a predefined conceptual interpretation. The interviews were designed to facilitate spontaneous and individually meaningful descriptions. Additional open-ended follow-up questions were asked to encourage elaboration and clarification when needed (e.g., “When you said ‘xx,’ what did you mean?” or “You mentioned xx. Could you tell me more about it?”). Each interview lasted between 45 and 60 minutes.

### Analysis

In line with Giorgi’s rigorous method, each step of the analysis required the adoption of a phenomenological attitude. As the responsible analyst, the first author sought to suspend prior assumptions through phenomenological reduction, allowing the phenomenon to be approached as it presented itself to consciousness. This attitude was critically discussed among the research team. The research team comprised one doctoral researcher (first author) and two experienced phenomenological researchers (third and fifth authors), enabling ongoing methodological guidance and reflexive discussion throughout the analytic process.

#### Giorgi’s descriptive analysis

In this study, the term “lived experience” refers to the pre-reflective, embodied, and meaningful way in which chronic migraine is experienced from the first-person perspective. Rather than referring simply to events or subjective reports, the term describes how the condition is lived through the body and shapes everyday life.

Analyzing the data requires a careful, systematic five-step process (Fig. 1). To better understand the process, each step is explained in detail.

**Fig. 1:**
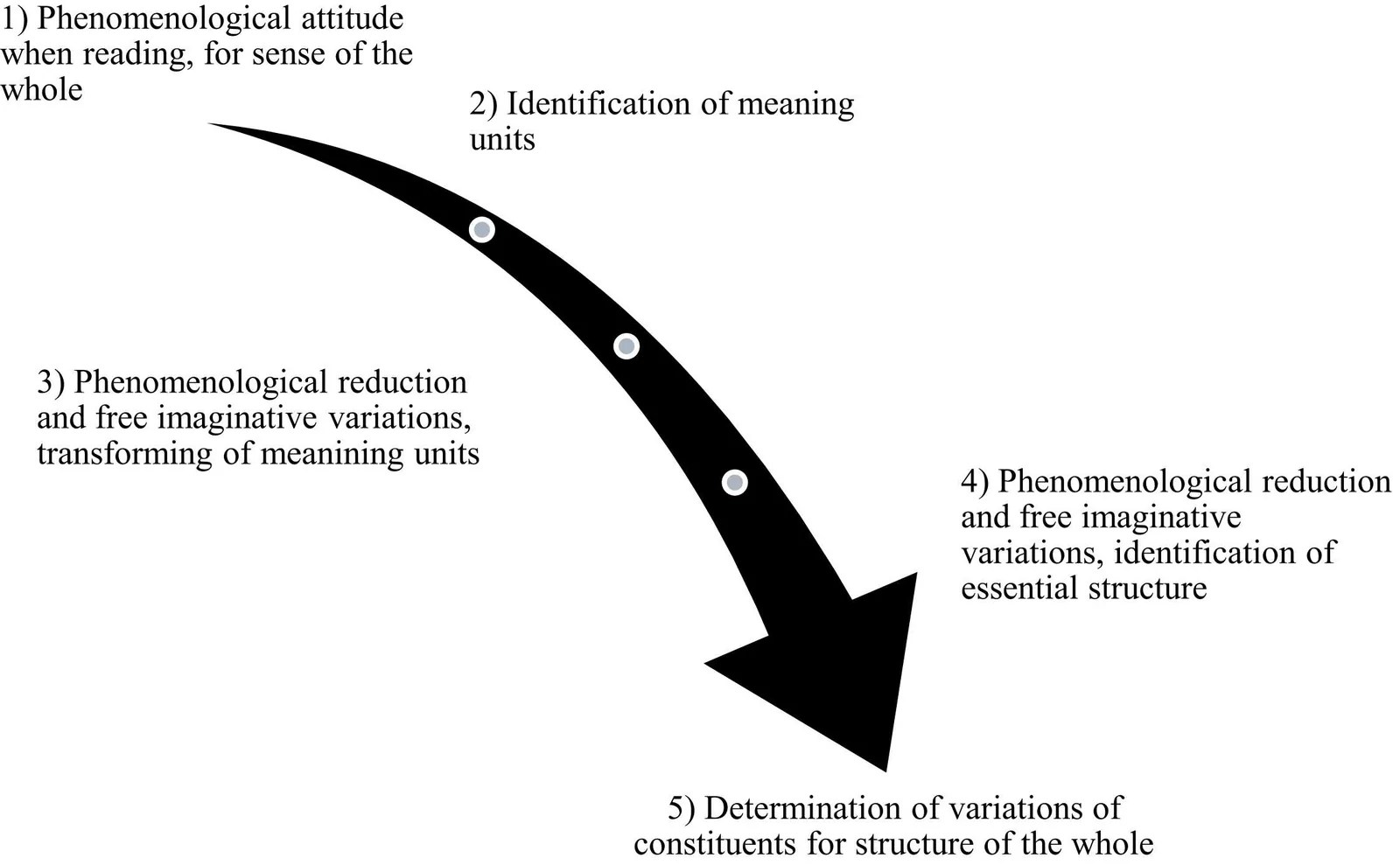
Giorgi's Five-Step Analysis.

**Reading for the Whole.** Following Giorgi's analytic framework (2009, p. 128), the first author read each transcript several times to understand each participant's account as a coherent whole before identifying meaning units. Throughout this process, the first author sought to maintain the phenomenological attitude by applying the phenomenological reduction. This involved deliberately setting aside, as far as possible, prior theoretical knowledge, clinical experience, and personal assumptions to focus on how participants described their experiences of chronic migraine. Conducting the interviews personally enabled the researcher to remain closely connected to participants’ lived meanings and to immerse themselves in the experiential context of the data throughout the analytic process.

**Identification of Meaning Units.** In line with Giorgi (2009, p.129–130), the transcripts were delineated into meaning units, defined as segments of the descriptions that reflect a shift in meaning, as experienced by the researcher. This segmentation remained faithful to the natural flow of the participant’s embodied experience.

An example of a meaning unit is as follows:

And then it starts in my head, you know, this sort of anxiety about my own body. Like, now, you know, an attack is coming. In a way, I go into a really panicked state. My heart starts pounding, and this feeling of anxiety comes over me, this sense of, oh no, now this terrible pain is coming. How am I going to survive it?

**Transformation of Meaning Units.** Following Giorgi’s method (2009, p. 130–137), each meaning unit was transformed from the first-person perspective into the third person to emphasize the phenomenon itself, rather than the individual subject. This was achieved through a method called “free imaginative variation” (Giorgi, 2009, p. 131–133), which helps identify the essential aspects of the experience. Free imaginative variation is a reflective process in which the researcher, with the help of intentional acts of consciousness, without being bound by strict rules or constraints, creatively removes or alters elements of the description to capture the intention of the experience. This process will take some time, and the researcher may need to write several versions before accomplishing the desired expression (Giorgi, 2009, p. 132).

The researcher’s phenomenological sensitivity, together with a phenomenological attitude, is required to identify expressions that reveal the lived meaning of the experience. These phenomenologically relevant expressions are segments of the data in which the participant’s description conveys the experience as it is lived, rather than merely reporting events or explanations. Each meaning unit was treated as expressing a distinct aspect of the lived experience and was subsequently transformed into phenomenologically sensitive language. The final achievement of this process is the discovery of the invariant meanings that each meaning unit offers as contributing to the total structure of the phenomenon being researched. The phenomenon would not retain its essential structure if any of the invariant constituents were removed.

The following example illustrates the transformation process used to determine invariant meaning. First, the participant's description is transformed into a third-person expression:

P explains how a kind of anxiety about her own body begins in her mind. She describes feeling that a migraine attack is coming. She is entering a panicked state, with her heart starting to pound and anxiety rising, accompanied by fear of severe pain and uncertainty about how she will survive it.

Second, the following example illustrates how the phenomenal characteristics of the description were clarified, making the meaning of the described experience more explicit:

P explains that anxiety intensifies at the same time as the migraine attack begins, leading to a panicked state. She describes a state of experience in which her heart beats more forcefully, and the fear of the pain increases with uncertainty about how she will survive it.

Finally, the following example illustrates how the description was transformed into its invariant meaning.

P experiences panic and anxiety at the onset of a migraine attack, accompanied by a stronger heartbeat and increasing fear of pain, with uncertainty about how she will survive the attack.

**Identification of the Essential Structure.** In the fourth step (Giorgi, 2009, p.194–195), an eidetic structure was derived through free imaginative variation of the transformed meaning units. An eidetic structure is the essential organization of a phenomenon as it is lived, comprising the meaning constituents necessary for the experience to be what it is. This organization involved systematically considering how aspects of the experience could be varied or removed and assessing whether the phenomenon would remain unchanged. Through this process, distinctions were drawn between essential and contingent features. The invariant essence thus constitutes the general structure of the phenomenon, encompassing those meaning constituents that are necessary and irreducible across variations in participants’ experiences.

**Determination of Variations of Constituents.** In the final step, the structure of the bodily experience of chronic migraine was used to deepen understanding of the data and the dynamic relationships among its constituents. Following Giorgi (2009, p.199–200), this step involves synthesizing the findings into a description of the essential structure of the phenomenon, while clarifying the interrelations among its constituents. In this way, the structure serves as an integrative expression of the unity of the concrete data.

## Results

In line with Giorgi’s method (Giorgi, 2009, p.195), individual descriptions of the fourth step (Fig. 1) were synthesized into a unifying structure that captures the invariant features of chronic migraine as experienced by eight participants. The findings are presented as constituents of a unified phenomenological structure of bodily experience in chronic migraine. The analysis aimed to identify invariance that emerged across participants’ descriptions, rather than distinct categories or types of experience.

### The synthesized eidetic structure of chronic migraine

According to Giorgi (2009, p. 199), this structure does not represent a philosophical or universal essence, but what phenomenological descriptive analysis refers to as an “eidetic-level invariant”*—*an eidetic type. In this context, the eidetic type refers to the essential structure of experience that remains constant across individual variations. It captures what is common and meaningful in how the phenomenon is experienced, rather than describing an abstract or metaphysical essence. In other words, it expresses the stable pattern of experience that allows the phenomenon to appear as what it is, despite differences between participants. The description of the unified structure of the phenomenon in the bodily experience of chronic migraine can be described as follows:

One type of bodily experience of chronic migraine took place in the context of persistent migraine attacks, and in situations in which the participants, P1–P8, perceived that unpleasant sensations interfered with their sense of bodily control, participation and agency. In such moments, the participant (P) experiences a fear of losing control over their body. This fear is preceded by heightened bodily vigilance and tension, as P anticipates the onset of an attack. As the migraine intensifies, P becomes increasingly aware of a desire to withdraw, to retreat to a private space in an effort to calm down and alleviate their overwhelming sensations. Over time, as the persistent nature of the attacks remains unchanged, P comes to recognize a profound sense of exhaustion, which continues to shape their lived experience of chronic migraine. In response to these enduring and overwhelming experiences, P gradually strives to develop personal strategies to calm the body, manage pain, and regain a sense of stability.

Building on this eidetic type, four key components were identified as essential to the phenomenon: sense of agency, sense of bodily control, sense of bodily exhaustion, and sense of adaptation. These components were present in every participant’s account and were therefore considered necessary for the phenomenon to manifest (Table 2).

**Table 2:** Key constituents of general structure and empirical variations experienced by each participant.

	Sense of Agency	Sense of Bodily Control	Sense of Bodily Exhaustion	Sense of Adaptation
PP1	Disruptions in agency when acknowledging migraine as a normal state of life with no sense of well-being.	Loss of bodily control when the connection to the body shut down during attacks.	Embodied exhaustion related to constant fatigue, debilitating neck pain, and poor sleep recovery.	Adapted strategies for tolerating pain and distancing from bodily discomfort.
PP2	Diminished agency related to unpredictable bodily sensations due to anticipating attacks.	Diminished bodily control when cognitive functions such as thinking, speech and focus were impaired during attacks.	Embodied exhaustion related to ongoing headaches, muscle tension and depression.	Adapted strategies for calming the body through physical exercises and treatments.
PP3	Fluctuating agency as migraine dictated the rhythm of daily life and limited chance for action.	Restricted bodily control related to limited movement during attacks.	Embodied exhaustion related to persistent fatigue, social guilt, muscle tightness and inadequate sleep.	Adapted strategies for tolerating pain and detaching from pain, calming the body and distancing from discomfort.
PP4	Reduced agency related to constant bodily monitoring and hypervigilance driven by fear of attacks.	Diminished bodily control when brain fog and exhaustion overtook functioning during attacks.	Bodily exhaustion related to hyperarousal, nightmares, fatigue and irritability linked to fear of attacks.	Adapted strategies for tolerating and managing pain and distancing from physical discomfort.
PP5	Compromised agency when fear of movement-induced attacks dominated daily life.	Loss of bodily control when swallowing and breathing difficulties were heightened during attacks.	Embodied exhaustion related to ongoing hyperarousal and panic responses linked to fear of attacks.	Adapted strategies for calming the body through physical exercises and treatments.
PP6	Diminished agency related to traumatic life events and heightened bodily stress.	Limited body control when exhausting pain dominated functioning during attacks.	Embodied exhaustion related to swallowing, breathing difficulties, stress, fatigue and coexisting illnesses.	Adapted strategies for calming the body through physical exercises and treatments.
PP7	Reduced agency related to a hectic lifestyle and continuous bodily overload.	Diminished bodily control related to dizziness and incapacitating headaches during attacks.	Embodied exhaustion related to persistent pain symptoms and constantly having to struggle.	Adapted strategies for calming the body through physical exercises.
PP8	Loss of agency when bodily control was lost during attacks.	Disconnection from bodily control when the left side of the body became paralyzed during attacks.	Embodied exhaustion related to ongoing fear of attacks and bodily pain.	Adapted strategies for grounding and calming the body.

### Bodily experience of chronic migraine: a representation of empirical variations

Table 2 summarizes the most invariant meanings, or key components, of the general structure of the bodily experience of chronic migraine, while also illustrating the empirical variations that emerged in participants’ descriptions. Participant quotations were not treated as self-evident descriptions, but as expressions of experiential meaning, phenomenologically clarified while remaining faithful to participants’ experiences.

Drawing on the insights provided by the general structure and its key constituents, the following section offers a more detailed account of how the participants meaningfully experienced each essential component.

#### Sense of agency

"Sense of agency" is defined as the pre-reflective experiential sense of "I can," referring to the lived potentiality for action. It denotes the immediate sense of oneself as capable of moving, acting, and engaging with the world through the lived body.

Participants' experiences of agency were often disrupted by the persistent and unpredictable nature of migraine. For example, P1 described difficulty distinguishing between migraine-related sensations and their baseline bodily state:

I don’t know what my own normal is. What is part of a migraine attack, and what isn’t, in a way? Moreover, as healthy as a person can be, but in a general sense, a kind of healthy, balanced bodily state. I really don’t know. I have no point of comparison.

And P4 reflected on how migraines seemed to take control over their lives, leading to frustration and despair:

I feel irritated that I let these things affect me so much. It frustrates me that I allow this illness to define my life too much. Why do I give it so much power? It irritates me, but it also, at times causes despair—like, why does it hurt again?

For several participants (P2, P4, P5, P6) the unpredictability of attacks led to a heightened state of vigilance and a diminished sense of agency. Life outside the home was often characterized by hypervigilance, panic, and escape-oriented behaviors:

I observe my surroundings, considering what in this environment might trigger my migraine and what might not cause it. Should I really do this or that if there's a chance it might lead to a migraine? (P4)

I have this flight reaction. I’m not sure if it’s related, but I have a strong flight response. It’s so intense that my body goes into hyperactivity, almost like there’s nowhere to go. A new house, a new space. I don’t do it consciously, but I already know where I’d escape if something happened. Wherever I go, I’m not exactly afraid, but my brain automatically scans for the exits. (P5)

P8 experienced the sense of agency as a loss of control when their symptoms signaled the onset of an attack:

It feels like, in my body—and I think I’ve tried to describe it like this before—kind of like…. How should I put it? A bit like I have no control over my body. In theory, I would really want to get out of that curled-up position during a migraine attack and just be my normal self again. But in a way, I can’t control it myself. It feels as if someone else is steering my body, not me. Yeah, it’s really…. It feels overwhelmingly vast, like I can’t control it at all. Someone else is in control. No matter how much I want to stop everything that’s happening in my body during an attack, I just can’t.

P6 and P7 described a compulsion to remain active during attacks, even when rest was medically advised. This behavior reflected the need to struggle to maintain agency in the face of debilitating symptoms:

In principle, I know that, for example, during a migraine, I really shouldn’t do much of anything. You're not supposed to do anything then. But I stubbornly go out for a walk and just push through, crying when I have to. I go. I just go. I’m kind of like that. I don’t really know how to…. I think I’m afraid of falling apart and ending up stuck in bed. I know I should show myself some compassion in those moments. (P6)

But still, I worked, even though I was on sick leave for a few weeks at a time – just short periods, like sick leave. I did remote work, which made it possible for me to keep going. Or should I have just not worked at all? I've wondered about that sometimes, too. Would I have recovered sooner that way? (P7)

Several participants (P2, P5, P6, and P8) reported needing hospital care due to the severity of their symptoms and the overwhelming diminishing sense of agency:

And then, my friends immediately took me to the emergency room, where I stayed overnight and received medication. That’s when it was essentially diagnosed, that I was having these kinds of severe migraine attacks that require help, because there’s no physical control over the body. After that, I actually had a more stable period, during which the migraines continued, but were more manageable. (P5)

#### Sense of bodily control

“Sense of bodily control” refers to the experienced capacity to initiate, regulate, and sustain movement. It describes the lived sense of manageability and coordination, i.e., how movement is experienced as either fluidly available or increasingly difficult to organize and maintain. Participants often felt that their sense of bodily control was diminished, and—particularly when anticipating an attack—the body became unpredictable and unreliable.

Several participants (P2, P4, P7) reported a fear of losing motor control before the onset of symptoms. As the attack progressed, panic and immobility intensified, and cognitive functions such as thinking, speaking, and focusing became impaired (P1, P2, P4, P5, P6, P7, P8).

P1, for example, described how during an attack, their body entered a state of shutdown:

It’s as if the body enters a state where it’s not exactly locked, but kind of numb. The brain knows that something is off, that I’m not well. I can’t do anything, but I don’t really know what’s actually happening, aside from, of course, the headache. But there’s something mysterious about it because it’s like the connection to everything else is severed. I’m still here, but then something is happening.

Similarly, P2 noted how their speech and thought processes were affected:

If I get a really intense migraine attack, it affects my ability to think. I lose words, and it’s hard to find them. My speech becomes a bit slurred, and I struggle to focus.

Severe pain and vomiting could render some participants immobile:

So, when it [pain] has left the right side, a squeezing feeling starts, like you have a ring around your head. And then someone just keeps tightening it more and more, making the ring around your head tighter. And then there might be these spikes inside the ring. The spikes pierce into my head. And then it just keeps tightening and tightening, and then it’s, like, now I can’t do anything. (P7)

Such an episode often ends nowadays with me vomiting, and then I can't move because I have no balance…. Somehow, it is related to the sense of balance, that if someone even moves in the bed, I might vomit*.* (P3)

P8’s kinesthetic sensitivity and coordination were disrupted:

Whenever an attack is coming, I kind of forget. Like, oh no, am I getting some new illness, or is this just part of my condition? For example, fine motor tasks with my left hand become increasingly difficult, and activities like walking up and down stairs start to affect my coordination. (P8)

#### Sense of bodily exhaustion

“Sense of bodily exhaustion” is understood as a pre-reflective modification of lived embodiment, characterized by a diminished or constrained availability of energy, responsiveness, and openness to action. It is not a failure of control, but a mode of being-in-the-world, referring to how the body shapes one’s possibilities for action, engagement, and everyday participation, in which activity becomes effortful and is often interrupted or postponed.

For participants, living with chronic migraine involves a continuous struggle against physical fatigue and exhaustion. Participants described exhaustion not as a temporary state, but as a persistent condition that threatened their ability to manage work, leisure, and recovery.

A sense of exhaustion was deeply embedded in participants’ everyday lives. P1 described fatigue as the most dominant and disabling aspect of their condition:

My primary problem is the fatigue my body experiences. We've discussed this with my doctor, and I’m sure I could get the controversial diagnosis of chronic fatigue syndrome if I insisted. However, it doesn't affect my situation like that. From my bodily perspective, fatigue is my biggest problem at the moment because I can't manage to do anything.

P4 similarly emphasized the overwhelming nature of fatigue:

Yes, the exhaustion is also significant. Sometimes, it’s so overwhelming that it feels like I could fall asleep immediately. If I just closed my eyes, I could fall asleep right away. The migraine causes that.

For some, the ongoing nature of migraine led to a sense of despair, as it drained their energy and cognitive capacity:

It's [migraine] still debilitating, even though I don't get those severe attacks very often. It still affects my ability to think, impacts my sleep, and affects my energy levels. (P2)

Every participant reported persistent stiffness, both conscious and unconscious, in their neck and shoulder muscles, which contributed to their overall fatigue, for example:

That fatigue easily goes to my head, and the tiredness also causes headaches. It can even trigger a migraine. It tends to happen quite quickly. I believe that when I’m tired, I tense my shoulders even more. I feel many sensations in my neck and shoulders, like tension, cold and all sorts of things. It’s often been like this, and it makes me more vulnerable to migraines. (P3)

In addition to physical fatigue, some participants (P2, P3, P4, P7) described emotional exhaustion and social guilt because of their inability to meet expectations or participate in social life. As P3 explained:

It even makes me feel guilty that many social situations are difficult when there are some things I just can't do.

#### Sense of adaptation

*“*Sense of adaptation” is understood as a situated and ongoing reorientation of embodied engagement with the world in response to fluctuating bodily conditions. It describes how individuals modify their modes of living and acting in response to changing constraints.

Participants described various ways in which they adapted to living with chronic migraine. They used physical exercise and therapeutic treatments to help regulate their bodies and enhance their sense of control (P2, P5, P6, P7). Interestingly, pain was not always perceived as the most distressing symptom. Taking medication early enough was reported to reduce the intensity of attacks and promote coping:

In my case, it’s not extreme pain, like some other migraine sufferers really experience. I can imagine what that's like, but it's still temporary. Even though it dominates things all the time, I’m not constantly in terrible pain. (P1)

I don’t even see the pain as the worst symptom. Of course, the pain is terrible and uncomfortable, but medication helps the pain; it alleviates it. However, it doesn’t alleviate the other symptoms*.* (P4)

The home environment provided a secure setting in which the participants could manage their symptoms. At home, they felt a sense of relief following an attack, even though the exhaustion remained, as P1 explained:

Still, it frees you, but then, it also takes you to the couch from the perspective of tiredness. Then you have this heavy feeling and can’t do anything. It’s not just that. The mind really wants to do a lot, but you feel sort of sluggish.

And P2 added

It also depends to some extent on where I am. Like, if I’m at home, it usually goes like this: After I’ve thrown up a couple of times, it kind of passes and the headache starts to ease.

Adaptation also involved strategies such as forgetting about attacks or consciously distancing oneself anticipating the next attack:

Of course, when there are such long periods, my thoughts easily go there. That it won’t bring it on yet, and it causes unnecessary tension, making it a horrible experience. However, it’s also true that you forget it quite quickly before the next one comes, and that's what you want. (P3)

Some participants described a growing trust in their body’s ability to manage the situation, even when they still felt uncertain:

Because I notice that the body can manage the situation quite well, but when the attacks are still too close, the panic comes from whether I can control it. However, in the background, they have become less frequent. But I want to have the upper hand. (P5)

Grounding techniques—some of which were discovered unconsciously—were also described as beneficial for regaining a sense of presence and calm, for example:

And then I got this feeling like, darn it, I’m the one in charge of my body, not anyone else. So, I planted my feet firmly on the ground. Then, I somehow just focused on that, like, I have the floor beneath my feet. Nothing is actually happening to me right now. After that, I felt this sense of calm and serenity. It was pretty cool and a really amazing feeling. Something I’d like to experience more often. A strong feeling, like anxiety and fear, just lifted from my upper body and chest. And I felt sort of free. (P8)

Together, the four key constituents discussed above form a cohesive whole that captures the essence of the bodily experience of migraine. However, the constituents are not as distinct as they might appear in Table 2. They are analytically separated to clarify different aspects of the phenomenon. Consequently, understanding the phenomenon depends not only on the individual constituents, but also on how they relate to one another. If one constituent were removed, the essential structure of the experience would no longer adequately represent the phenomenon.

Each constituent must therefore be understood in relation to the others, as they constitute the experiential lifeworld with chronic migraine. They are not isolated elements, but interwoven aspects of how the participants navigate their daily lives when they have to live with a chronic and often unpredictable condition. The loss or disruption of agency and bodily control is frequently accompanied by profound fatigue and emotional strain. Yet, the participants also described their capacity to adapt and develop personal strategies to manage their symptoms, reclaim their bodily presence, and maintain a sense of continuity in their lives. This phenomenological structure offers a nuanced understanding of chronic migraine as an embodied experience—one that transcends clinical symptoms to encompass the existential, relational, and adaptive dimensions of everyday life.

The following section further contextualizes and discusses these insights in relation to the existing literature and theoretical perspectives. As such, the descriptive phenomenological analysis serves as the foundation for a deeper engagement with the meaning of chronic migraine. Importantly, the identified structure is also shaped by migraine-specific temporal features, including the cyclical nature of attacks and the anticipatory experience preceding them. This temporality contributes to a persistent state of bodily vigilance and influences how participants relate to their bodies between and during attacks.

## Discussion

This study identifies a complex, interrelated structure comprising four essential constituents that shape the bodily experience of chronic migraine: sense of agency, sense of control, sense of exhaustion, and sense of adaptation. In line with Husserl’s phenomenology and Giorgi's method, these constituents describe the body as the primary mode through which the world is experienced and acted upon. They should not be understood as separate or mutually exclusive categories, but as interrelated and overlapping structures of meaning that together constitute a unified experiential whole. Their interrelatedness reflects the intrinsic structure of lived experience. Together, these constituents reflect not only the embodied burden of chronic migraine, but also its existential and relational dimensions as they are lived in everyday life.

Participants’ accounts revealed how the unpredictability of migraine attacks disrupted their sense of bodily control and agency. This was lived as a state of heightened hypervigilance, anticipatory anxiety, and a sense of alienation from their own bodies. At the same time, participants described how adaptation, bodily awareness, intuitive self-regulation, and self-care practices enabled them to reclaim a sense of agency. Their experiences demonstrate that living with chronic migraine involves a continuous negotiation between vulnerability and agency, loss and reconstitution of bodily control.

These experiences of unpredictability and heightened vigilance resonate with existing accounts of the underlying neurobiological mechanisms of migraine, such as altered sensory processing, central sensitization, and hypothalamic dysregulation (Goadsby et al., 2017). However, rather than reducing these experiences to underlying mechanisms, the present findings highlight how such processes are lived and given meaning at the level of embodied experience. In this sense, the experienced loss of bodily control may also be understood as reflecting a disturbance in the anticipatory and regulatory dimensions of being-in-the-world.

### Sense of agency and bodily control towards bodily trust

A central finding of this study is the role of the sense of agency in shaping how participants confronted bodily trust in daily life. Participants experience the unpredictability of migraine as destabilizing both their sense of bodily control and their sense of agency, giving rise to heightened vigilance, anticipatory tension, and, at times, a sense of estrangement from their bodies. According to McGinley et al. (2025), challenges to stabilizing their sense of agency are threatened by symptoms that may be present during interictal periods in the migraine cycle.

At the same time, participants described becoming increasingly attuned to their experiences, and the interview situation itself appeared to support processes of reflection that brought new insights into their condition. Through these processes, participants seek to make sense of sensations in relation to their intuitive responses, at times identifying moments of clarity and even positive experience. These descriptions suggest that the sense of agency is not simply lost, but can be gradually reconstituted through embodied reflection and attunement.

Previous qualitative research, such as that by Palacios-Ceña et al. (2017), has shown that migraine pain can come to dominate everyday life and demand ongoing self-regulation. In line with this, the present findings highlight how disruptions in agency become particularly visible in situations where movement and intentional action are constrained.

Pain dominates the lives of individuals with chronic migraine and demands high levels of self-control. As Gallagher (2000) suggests, agency is inherently tied to intentional bodily movement, and its disruption becomes most evident when the body no longer readily supports action. Thus, in line with Zahavi (1994), the possibility of mobility will be threatened by a disrupted kinesthetic system.

Nevertheless, participants sought to make meaningful connections between sensations and their intuitive responses throughout the interview. They also identified moments of insight—even positive sensations—suggesting that agency can be reconstituted through embodied reflection.

The distinction between being a body and having a body appeared as an ontological theme in the data. This resonates with Husserl’s concepts of “Leib” (the lived body) and “Körper” (the body as a physical object). Wehrle (2020) builds on Husserl’s distinction and argues that “being a body” refers to the pre-reflective experience of existing in the world, in which the body functions as the medium of perception, action, and presence. In contrast, “having a body” becomes prominent in situations in which the body is experienced as an object of consciousness, such as during moments of illness or pain.

Participants described a shift from an unreflective, embodied immersion in daily life to a state in which the body becomes intrusive and disruptive. This dynamic interplay between being and having a body offers a meaningful framework for understanding how chronic migraine transforms the lifeworld and embodiment of those it affects. This tension between vulnerability and agency underscores the existential weight of chronic migraine as more than a neurological disorder; it is a condition that reconfigures one’s being-in-the-world. Participants described moving between being a body—a seamless, taken-for-granted mode of existence—and having a body that becomes an object of concern. The migraine-affected body was no longer experienced as a seamless part of the self, but rather, as something to be managed, endured, or tolerated. The existential tension between being-in-the-world and being constrained by one’s body highlights chronic migraine as a condition that reshapes the foundations of lived existence.

### Bodily exhaustion: Existential and social dimensions

The sense of exhaustion emerged as a pervasive and deeply existential aspect of chronic migraine. Participants described exhaustion not merely as a symptom, but as a persistent mode of being that shaped their daily lives, relationships, and self-perception. This resonates with broader literature that conceptualizes fatigue in chronic illness as a lived, embodied condition (Karsan and Goadsby, 2021, Kumar et al., 2023, Åsbring and Närvänen, 2002)). Exhaustion appeared across participants’ private, social, and professional contexts and was often accompanied by feelings of stigma and misunderstanding. This is highlighted with a review by Battista et al. (2023) and Rutberg and Öhrling (2012), who observed that individuals with migraine frequently report feeling stigmatized due to others’ limited understanding of the condition.

According to Peters et al. (2005), pain itself was not always described as the most distressing aspect of migraine, whereas Rutberg and Örhling (2012) emphasize that a migraine is more than a headache; pain together with other symptoms involves a temporary loss of control that makes it impossible to function normally. Rather, participants spoke of the cumulative presence of fatigue, cognitive fog, and the fear of losing control as intensifying their suffering. Constant vigilance and the anticipation of attacks were experienced as part of a cycle of tension and exhaustion, which further blurred the boundaries between illness and self.

Theoretical perspectives from Fuchs (2012) and Zahavi, 1994, Zahavi, 2003, Zahavi, 2018 underscore the role of body memory and pre-reflective self-awareness in shaping bodily experience. These ideas were reflected in participants’ descriptions of the prodromal phase, which they experienced as a meaningful window for recognizing and responding to early symptoms. Some participants expressed uncertainty in how to interpret or act upon these early sensations. Persistent tension in the neck and shoulders was described as interfering with their ability to attune to these signals. This aligns with Carvalho et al. (2014), who found that neck pain-related disability tends to occur with increased migraine frequency. In this context, the body’s memory of pain and its temporal unfolding were experienced as central to how chronic migraine is lived, understood, and navigated. In this sense, the experience of time becomes reconfigured, as the body carries forward traces of previous pain while anticipating its recurrence.

### Adaptation and embodiment: Pathways to reconstituted agency

Amidst bodily disruption and exhaustion, participants described processes of adaptation through which they sought to re-establish continuity and stability. These adaptive responses were not purely cognitive, but deeply embodied, guided by intuition rather than prescriptive routines. Participants spoke of adjusting their environments, regulating sensory exposure, and responding to early bodily signals. This resonates with Merleau-Ponty’s (1962) notion of the body as an active, intentional subject that seeks equilibrium, even in illness.

Adaptation was experienced in relation to a sense of safety within bodily and spatial contexts. Participants described how a greater sense of control in safe environments was associated with more manageable pain. They also spoke of moments when medication, adaptation, or even temporary forgetting of pain supported a renewed sense of bodily control. When the pain subsided, participants felt better able to reorient themselves toward self-regulation. Public spaces such as transport, gyms, and workplaces were often described as anxiety-inducing and as diminishing participants’ sense of control. This resonates with studies by Rutberg et al. (2013), which found that managing life to feel well while living with migraine involved striving to build a basic foundation of safeness by handling triggers and the symptoms of migraine.

These descriptions illustrate how participants´ lived space was redefined through the interplay of safety and threat. In contrast, the home was experienced as a safer space, where symptoms felt more manageable. This finding resonates with Ojala et al. (2014), who noted that chronic pain is often perceived as less intense in familiar and supportive environments. Despite these challenges, participants also described experiences of adaptation and the development of personal strategies to navigate symptoms and maintain continuity across different environments. These oscillations between safety and threat illustrate how chronic migraine reshapes one’s lived space and being-in-the-world.

Participants also described the importance of sleep, nutrition, and self-care in reconnecting with a sense of bodily control. As Weatherall (2015) and Martelletti et al. (2018) suggest, self-management and a positive orientation toward the body are meaningful in living well with chronic migraine. Similarly, Rutberg and Öhrling (2012) observed that individuals with migraine engage in both preventive and responsive strategies to support their well being while managing their condition. Participants noted that such strategies were not always accessible, particularly in moments of fear or loss of control. All participants emphasized the importance of cultivating a positive, intuitive relationship with their bodies through grounding, gentle movement, and environmental adjustments—embodied ways of reclaiming agency.

### Clinical implications and concluding reflections

Our findings suggest that collaborative migraine care would benefit from attending not only to symptom reduction, but also to individuals’ experiences and altered body awareness. Chronic migraine was experienced as disruptions in bodily control, heightened vigilance, uncertainty, exhaustion, and shifting relationships with the body in everyday life. Although these experiences were often difficult to articulate, they profoundly shaped participants’ sense of agency and their engagement with daily activities.

Participants also described a need to consciously reconnect with their bodies through supportive and embodied practices. These efforts reflected attempts to re-establish bodily trust, agency, and a workable sense of control in everyday life. Approaches focusing solely on symptom management may therefore overlook essential experiential dimensions of living with chronic migraine. This aligns with Emmenegger and Gantenbein (2025), who emphasize migraine as a phenomenological, subjective, and ontological event involving dimensions of suffering, consciousness, language, and human existence. Similarly, McPhee et al. (2022) highlight the importance of holistic approaches, while Varkey et al. (2013) and Estave et al. (2021) emphasize the need to incorporate patients’ perspectives into preventive and clinical practices.

Attending to individuals’ experiences may support more collaborative clinical encounters grounded in listening, validation, and sensitivity to how they perceive and understand their bodily cues in daily lives. In this way, phenomenological insight into embodiment can contribute to more person-centered care by recognizing the patient's capacity for more conscious embodied living.

### Strengths and limitations

This study focused on exploring the experience of women with chronic migraine, using a descriptive phenomenological approach. The participants were employed and living with chronic migraine, but did not have so burdensome comorbid conditions such as severe depression or musculoskeletal disorders. The decision to adopt this methodology was based on the aim of accessing and describing the essential structures of experience as they are lived, rather than interpreted or theorized. Although the findings are not intended to be generalized to all individuals with chronic migraine, they offer valuable insights into how the condition is embodied and experienced in everyday life.

The key methodological strength of this study lies in the use of Giorgi’s (2009) descriptive phenomenological method, which required the researchers to systematically bracket preconceptions and theoretical assumptions. This approach was chosen to ensure that the analysis remained grounded in participants’ descriptions and to minimize the imposition of external interpretations. Close adherence to the principles of phenomenological reduction, along with continuous critical reflection on presuppositions, enhanced the descriptive validity and trustworthiness of the findings (Giorgi, 2002).

In descriptive phenomenology, the researcher serves as the primary instrument of analysis, responsible for discerning and articulating the essential meaning structures of direct experience. From this perspective, it is methodologically coherent for the same researcher to conduct both the interviews and the analysis. Rather than seeking verification from participants, phenomenological research should adopt criteria that emphasize openness, reflexivity, and sensitivity to the meanings that arise during interaction between the researcher and participants (Lopes et al., 2025). Familiarity with the interview context may support more nuanced sensitivity to experiential meanings. Accordingly, the credibility of the findings rests on the rigor of the phenomenological reduction, the transparency of the analytical process, and sustained reflexivity throughout the research (Giorgi, 2002).

The participants in this study were employed working-age women, which may indicate a relatively high level of functioning and access to care. This may limit the transferability of the findings to individuals with more severe disability or to those with different socioeconomic circumstances. In addition, the recruited participants do not represent the full heterogeneity of chronic migraine experiences. Chronic migraine varies considerably in terms of attack frequency, symptom severity, duration of illness, and functional impact. The experiential structure described in this study may therefore not reflect the full range of embodied experiences associated with chronic migraine. Consequently, the findings should be understood as offering insight into the essential meanings of bodily experiences within this particular group of participants, rather than as statistically generalizable results.

The analysis focused on identifying the essential constituents of the phenomenon through eidetic variation, which was used to distinguish essential meanings from incidental features of the descriptions. These constituents may be described or named differently in other studies, but the focus here was on identifying the underlying meanings of the phenomenon. This approach aligns with Dahlberg’s (2006) view that phenomenological research seeks to uncover the “essence of essences” through careful reflection on meaning structures.

The novelty and contribution of this work are captured by two key factors. First, the use of a descriptive phenomenological method offers analytical depth beyond general qualitative designs, providing a systematic account of meaning as it appears through personal experience (Dahlberg, 2006, Giorgi, 2009, p.79). Second, the focus on embodiment brings attention to the sensory, affective, and relational dimensions of migraine that are not accessible through clinical measures alone. Together, these aspects support a deeper understanding of migraine as not only a neurological condition, but also a profoundly embodied and socially situated phenomenon.

One limitation of the study relates to the timing of the interviews. In several instances, participants had recently experienced migraine symptoms or were in a recovery phase during the interview. Although participants described migraine as an ongoing disruption in their lives, no severe attacks occurred during the interviews. This was an anticipated challenge, given the nature of the condition and was addressed by allowing participants to take breaks and by maintaining a flexible interview structure. Nevertheless, it is possible that fatigue or cognitive strain may have limited the depth of some responses or led to the omission of certain experiences.

## Conclusions

This study combines clinical relevance with phenomenological depth by illuminating the subjective, pre-reflective, and partially implicit bodily experience of chronic migraine. Participants described an altered relationship with their bodies, marked by unpredictability, heightened bodily vigilance, and challenges to bodily trust and control. By attending to these embodied experiences, the study highlights that chronic migraine is not only a condition of symptoms, but also a disruption of being-in-the-world.

These findings are significant for both individuals living with chronic migraine and healthcare professionals. Greater attention to personal experience may support clinical encounters in which patients’ descriptions of bodily unpredictability, exhaustion, and fluctuating agency are more fully acknowledged. This may, in turn, contribute to more dialogical and person-centered approaches to care, in which treatment is not limited to symptom reduction, but also considers how patients navigate everyday life with a changing and sometimes unreliable body.

Future research could extend this phenomenological inquiry to other chronic conditions characterized by persistent and fluctuating symptoms, further exploring how embodied experience, bodily consciousness, and agency are lived across different illness contexts.

## CRediT authorship contribution statement

**Riku Nikander:** Writing – review & editing, Supervision. **Kitty M. Suddick:** Writing – review & editing, Writing – original draft, Supervision, Methodology, Conceptualization. **Marja-Liisa Sumelahti:** Writing – review & editing, Supervision. **Pirjo Vuoskoski:** Writing – review & editing, Writing – original draft, Supervision, Methodology, Formal analysis, Data curation, Conceptualization. **Mikko Patovirta:** Writing – review & editing, Writing – original draft, Visualization, Methodology, Investigation, Formal analysis, Data curation, Conceptualization.

## Ethical statement

Ethical approval for this study was granted on 7 June 2022 by the Ethics Committee of the Pirkanmaa Hospital District (Approval No. R22005). The study was conducted in accordance with the Declaration of Helsinki. Written informed consent was obtained prior to participation. All personal data were pseudonymized and accessible only to the first and last authors.

## Funding

The corresponding author has received a personal grant from the 10.13039/501100004325Signe and Ane Gyllenberg Foundation (Grant No. 6257).

## Declaration of Competing Interests

The authors declare no conflicts of interest with respect to the authorship or publication of this article. The corresponding author received personal funding from the Gyllenberg Foundation (Grant Number: 6257).

## Data Availability

The datasets generated and analyzed in this study consist of sensitive health-related interview data. In accordance with the ethical approval obtained and due to the nature of personal medical information, the full datasets cannot be shared publicly. Anonymized illustrative excerpts that support the findings are included in the article.
